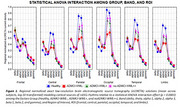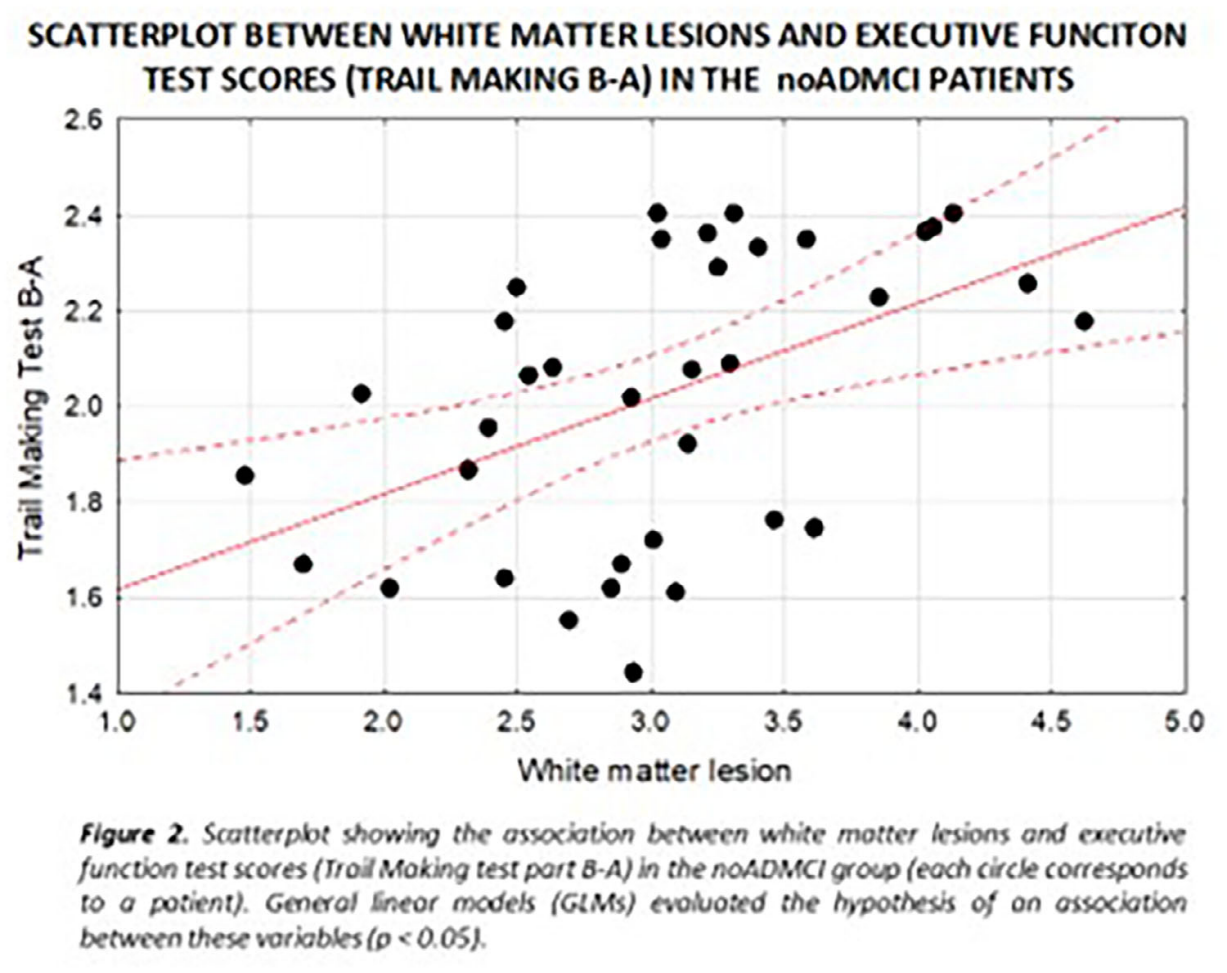# Abnormalities of resting‐state eyes‐closed electroencephalographic rhythms are not affected by white matter lesions in patients with mild cognitive impairment due and not due to Alzheimer's disease

**DOI:** 10.1002/alz70856_106342

**Published:** 2026-01-11

**Authors:** Roberta Lizio, Claudio Del Percio, Susanna Lopez, Mina De Bartolo, Matteo Carpi, Antonio Pio Afragola, Giuseppe Noce, Raffaele Ferri, Bahar Güntekin, Görsev Yener, Claudio Babiloni

**Affiliations:** ^1^ Sapienza University of Rome, Rome, Italy; ^2^ Oasi Research Institute – IRCCS, Troina, Italy, Troina, Italy, Italy; ^3^ Sapienza University of Rome, Rome, Rome, Italy; ^4^ Sapienza University of Rome, Roma, Italy, Italy; ^5^ Sapienza University of Rome, Roma, Rome, Italy; ^6^ IRCCS Synlab SDN, Naples, Italy; ^7^ Oasi Research Institute ‐ IRCCS, Troina, Italy; ^8^ Istanbul Medipol University, Istanbul, Turkey; ^9^ Dokuz Eylül University, Balçova, Izmir, Turkey; ^10^ Izmir Biomedicine and Genome Center, Izmir, Turkey; ^11^ San Raffaele Cassino, Cassino, Italy; ^12^ Department of Physiology and Pharmacology, Sapienza University of Rome, Rome, Italy

## Abstract

**Background:**

Patients with mild cognitive impairment (MCI) typically show abnormal high delta (<4 Hz) and low alpha (8–12 Hz) rhythms measured from resting‐state eyes‐closed electroencephalographic (rsEEG) source activities as well as white matter lesions (WMLs) measured from magnetic resonance imaging (MRI). Here we tested the hypothesis that rsEEG rhythms may not deteriorate with the increase of WLMs in patients with MCI due and not due to Alzheimer's disease (ADMCI and noADMCI).

**Method:**

An international database provided demographic, clinical, and rsEEG datasets for cognitively unimpaired older (Healthy; N = 30), ADMCI (*N* = 64), and noADMCI (*N* = 36) participants. The rsEEG rhythms spanned individual delta, theta, and alpha frequency bands. The eLORETA freeware estimated cortical rsEEG sources. The international database also provided MRI datasets for the ADMCI and noADMCI participants. T2 and Fluid Attenuated Inversion Recovery (FLAIR) images estimated the WMLs.

**Result:**

The posterior rsEEG alpha source activities were lower in the groups of the ADMCI with a less increase of WMLs (ADMCI‐WML‐), ADMCI with a high increase of WMLs (ADMCI‐WML+), and noADMCI with a very high increase of WMLs (noADMCI‐WL++) compared to the Healthy group (*p* < 0.001; *Figure 1*). This effect was dramatic in the ADMCI‐WML‐ group, marked in the ADMCI‐WML+ group, and moderate in the noADMCI‐WML++. Furthermore, a positive association between the increase of WMLs and the worsening of executive function test scores (i.e., Trail making test part B‐A) was observed in the noADMCI group (t = 3.25, *p* < 0.005; *Figure 2*).

**Conclusion:**

These results suggest that neurophysiological brain neural oscillatory synchronization mechanisms regulating cortical arousal and vigilance through alpha rsEEG rhythms are not affected by white matter tissue damage in MCI patients.